# Urban waste to construction material: *Casuarina equisetifolia* cone ash in coating mortars

**DOI:** 10.1007/s11356-026-37628-9

**Published:** 2026-03-17

**Authors:** Mariana Almeida de Azevedo Pessôa, Gustavo de Castro Xavier, Marcela da Silva Luna Paravidino, Laimara da Silva Barroso, Niander Aguiar Cerqueira, Sergio Neves Monteiro

**Affiliations:** 1 Advanced Materials Laboratory, UENF - North Fluminense State University, Av. Alberto Lamego, 2000, Campos Dos Goytacazes, Rio de Janeiro, 28013-602 Brazil; 2 Civil Engineering Laboratory, UENF - North Fluminense State University, Av. Alberto Lamego, 2000, Campos Dos Goytacazes, Rio de Janeiro, 28013-602 Brazil; 3 Materials Science Program, IME - Military Institute of Engineering, Praça Gen. Tibúrcio, 80, Urca, Rio de Janeiro, Rio de Janeiro 22290-270 Brazil

**Keywords:** Bottom ash, Coating mortars, *Casuarina equisetifolia*, Sustainable building materials, Filler effect, Microstructural

## Abstract

This study investigates the potential use of *Casuarina equisetifolia* cone bottom ash (CC-BA) as an additional material in rendering mortars for wall and ceiling applications. A bibliometric analysis revealed a significant lack of research on the topic. *Casuarina* cones, widely dispersed in urban areas, pose a public maintenance burden but can be used as fuel, producing ash with potential applications in construction. Mortars were prepared with a 1:6 cement-to-sand ratio, incorporating 5 to 25% CC-BA by weight relative to the mass of cement, in addition to a reference mix (CPII-00). The materials underwent physical, chemical, and microstructural characterizations. Fresh-state testing indicated satisfactory workability up to 15% CC-BA, with CPII-10 yielding the best results. All compositions met the consistency index requirements, although water demand increased with higher CC-BA content. Calorimetry results highlighted CPII-05 and CPII-15 as thermally satisfactory, indicating accelerated hydration kinetics. Air content decreased with CC-BA addition, suggesting a filler effect. After 28 days, mechanical strength remained favorable up to CPII-15. Water absorption and void index increased with 20–25% CC-BA, while lower contents showed comparable or improved values relative to CPII-00. Microstructural analysis, particularly via micro-computed tomography (μ-CT), confirmed the filler effects of CC-BA, enhancing performance up to CPII-15. Overall, CPII-10 demonstrated the most promising balance of properties, with CPII-15 also suitable for the proposed applications.

## Introduction

The rising global demand for construction materials, driven by global population growth of nearly 2 billion people, is expected in the coming decades, primarily concentrated in Asian and African countries. Consequently, the global built-up area is projected to double between 2015 and 2050 (Olsson et al. 2025).

The increasing demands of contemporary industrial applications, combined with the proportionally accelerated consumption of natural resources, underscore the need to develop and evaluate innovative materials with lower environmental impact. Recent studies have explored several alternatives, including thermoactivated crushed demolition residue (TCDR) (Tavares et al. 2024), TiO_2_ combined with mineral additives (Lara et al. 2023), fly ash (Ma et al. 2025), plaster byproducts (Ribeiro et al. 2024), ceramic waste (Cherene et al. 2023), and ornamental stone processing waste (OSPW) (Moreira et al. 2022).

The mechanical performance of coating mortars is primarily determined by bond strength, which reflects the adhesive capacity between the mortar and the substrate. Among the essential properties, insufficient pull-off strength can lead to coating detachment (Haddad et al. 2020; Nascimento et al. 2025).

A bibliometric analysis conducted using the terms “*Casuarina equisetifolia*” and “Building materials” in the ScienceDirect, Scopus, and Web of Science databases (restricted to the fields of engineering, materials science, environmental science, and multidisciplinary studies within the period from 2015 to 2025) confirms the absence of studies. This gap justifies the present investigation to contribute data on the material’s physical, mechanical, and microstructural properties, particularly when applied in mortars for walls and ceilings.

**Fig. 1:**
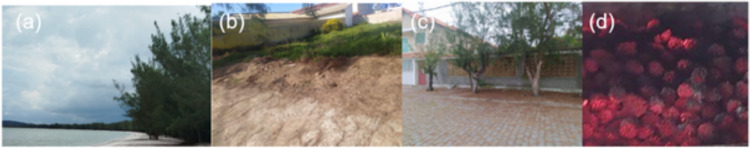
*Casuarina equisetifolia* **a** extension, **b** deposited in urban areas, **c** urban areas, and **d** firewood

Given the material’s widespread availability and dispersion in urban areas, *Casuarina* cones can even represent a cost burden for public authorities, as street sweeping services are required to remove them. Without proper disposal, their decomposition would take several years. These cones also exhibit woody characteristics similar to timber, making them suitable for use as fuel in small furnaces (Fig. 1).

## Bibliometric surveys

To quantify the scientific relevance of the topic, a bibliometric survey was conducted on September 24, 2025 (Table 1). The search term “*Casuarina equisetifolia*” was applied to the ScienceDirect, Scopus, and Web of Science databases, filtered by the fields of engineering, materials science, environmental science, and multidisciplinary studies, covering the period from 2015 to 2025**.**

**Table 1:** Search results for “*Casuarina equisetifolia*” on September 24, 2025

Databases	Results (2015–2025)
ScienceDirect	400
Scopus	198
Web of Science	96

**Fig. 2:**
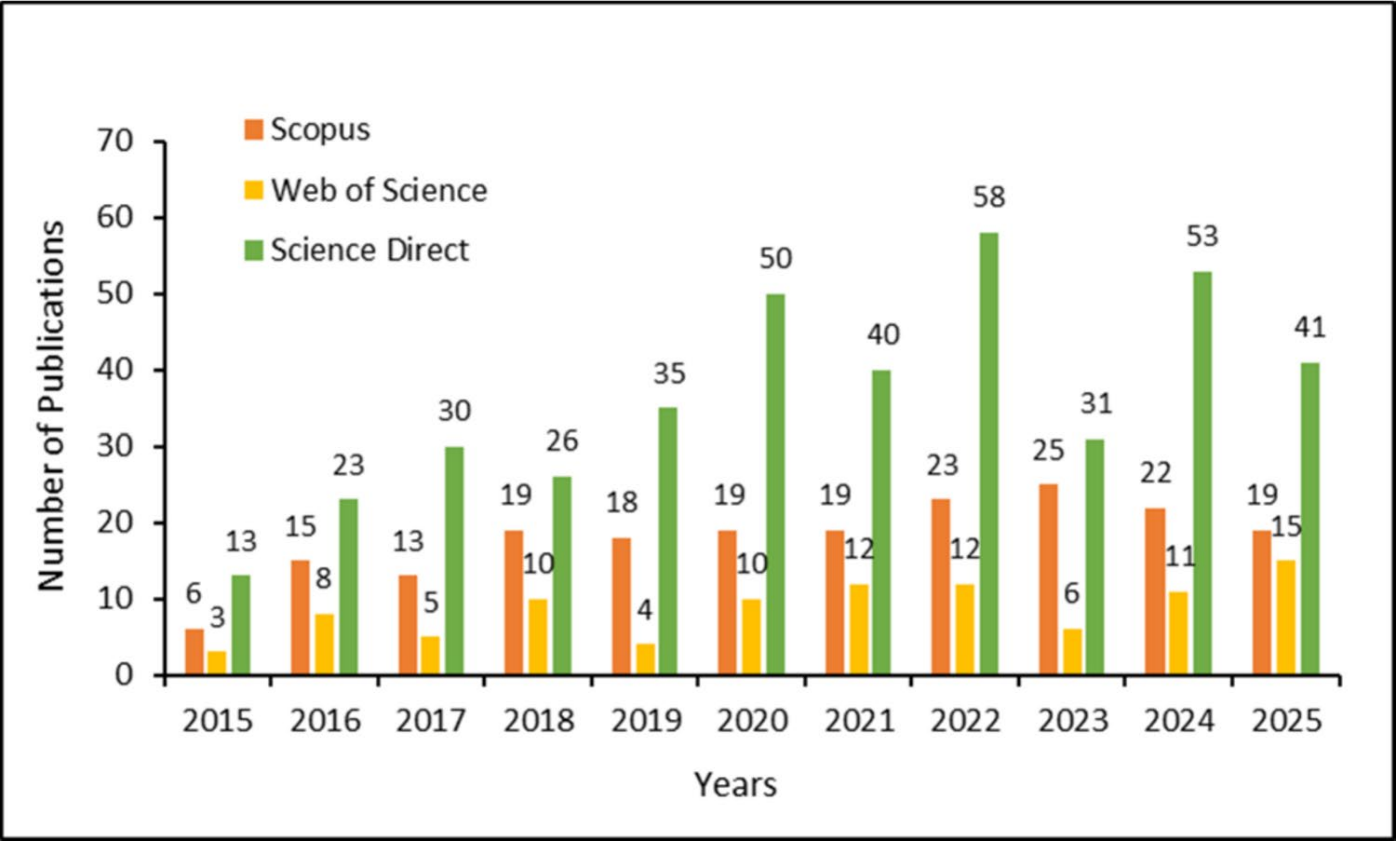
Graphical representation of the search results for the term “*Casuarina equisetifolia*” from 2015 to 2025

Figure 2 illustrates the annual distribution of publications; a slight decrease can be observed in 2019, followed by a modest increase in research on the topic in the subsequent years. No clear or justifiable explanation for this fluctuation has been identified.

**Fig. 3:**
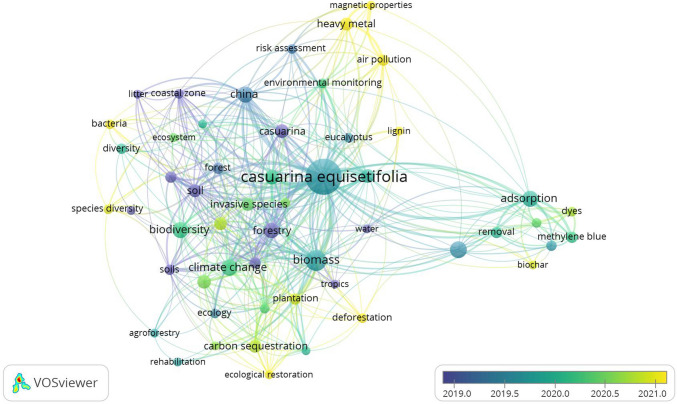
Network generated by the keyword “*Casuarina equisetifolia*”

For a better analysis among the researched articles, the VOSViewer software was used to build a network between the keywords addressed in the journals (Fig. 3). Based on the keyword network generated from the articles retrieved using the term “*Casuarina equisetifolia*,” with a minimum keyword occurrence threshold of seven, Fig. 3 shows that a noticeable concentration of connections is observed around the term “adsorption,” which reflects the ongoing research focus on the use of activated carbon as an adsorbent material. Notably, a higher concentration of studies emerges from 2020 onwards, indicating a recent increase in research focused on *Casuarina equisetifolia*. However, there remains a clear absence of key terms directly related to civil construction, construction materials, and mortars, such as cement, hydration, concrete, porosity, and mechanical properties, thereby highlighting a significant gap in the literature within this domain.

**Fig. 4:**
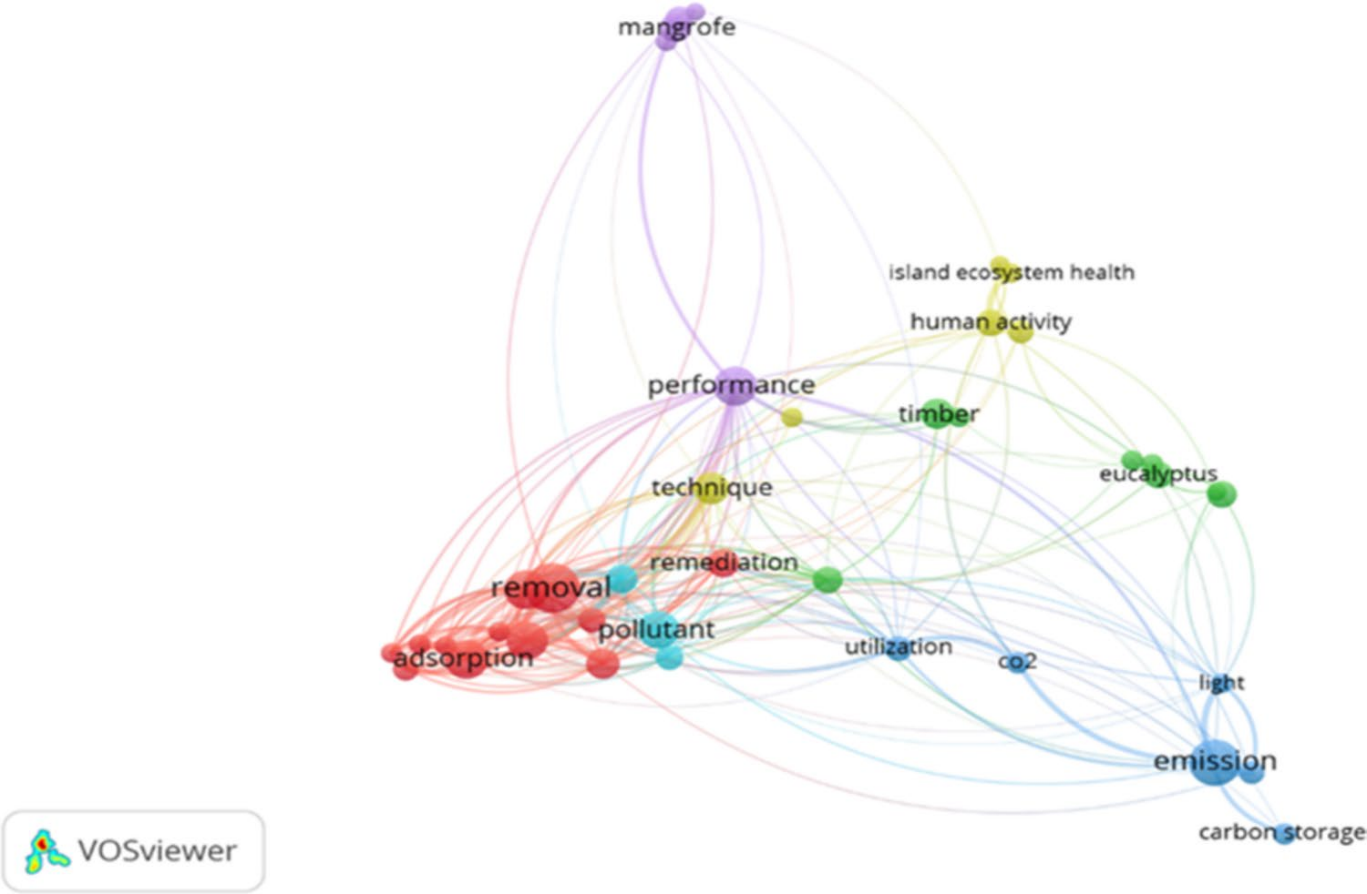
Network of the combination of the keywords “*Casuarina equisetifolia*” and “*Building Materials*”

When the keywords “*Casuarina equisetifolia*” and “Building Materials” are incorporated into the research scope, while applying the same search parameters and databases, an even more pronounced limitation in the breadth of the subject matter becomes evident (Fig. 4), with 37 results in ScienceDirect and 2 results in Scopus. The use of broader keywords highlights a significant gap in the literature regarding the study of *Casuarina equisetifolia*, particularly concerning its application in building materials.

## Materials and methods

**Fig. 5:**
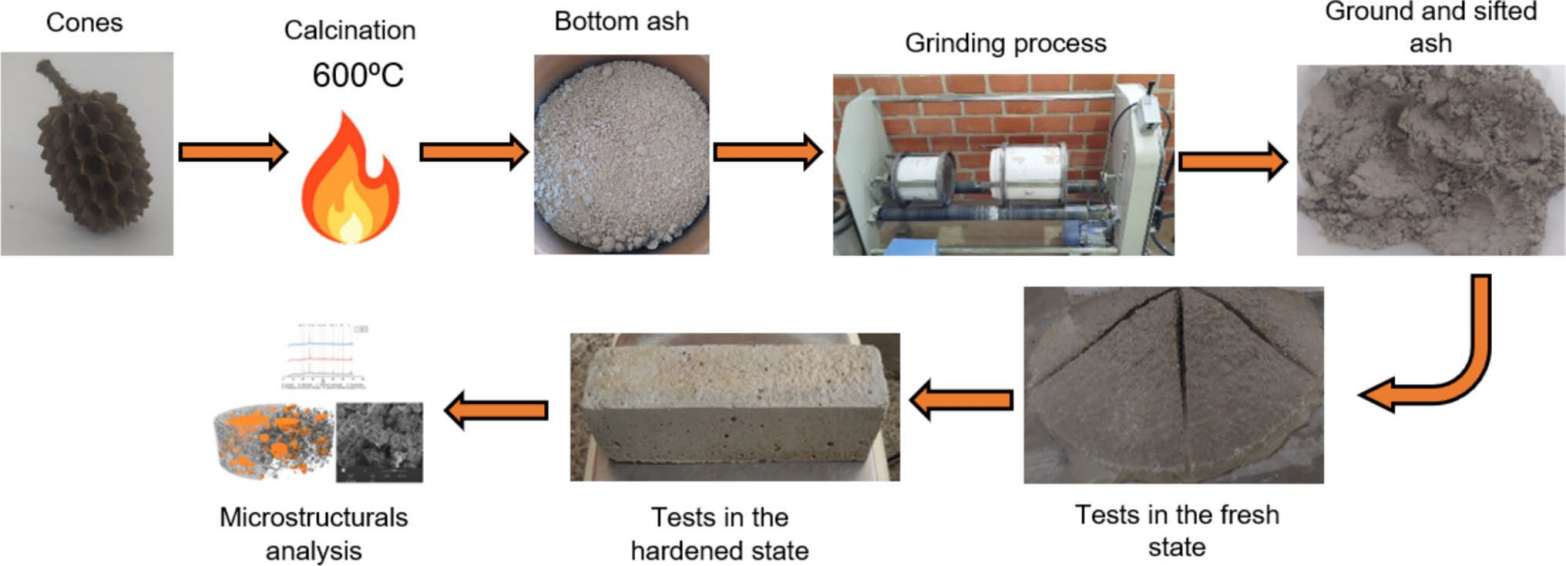
Summary of methods

The CC-BA, calcined at an adjusted temperature of 600 °C in a muffle furnace and ground for 1 h, was subsequently sieved through a 200# mesh (Fig. 5). Additionally, blended Portland cement with limestone filler (CPII-F) equivalent to Type IL (ASTM 2024) and CEM II-L (CEN 2011) cements, sand, and water were used. After this preparation of the materials, the mortars were prepared for conducting the tests in the fresh and hardened states, as well as for microstructural analyses.

The mortars were prepared using a 1:6 mixture (cement to sand) by adding CC-BA at levels of 5%, 10%, 15%, 20%, and 25% relative to the cement mass (Table 2). The mixtures were proportioned with fixed cement and sand contents, while the ash was added in increasing percentages by volume to ensure precise dosage control and experimental reproducibility.

**Table 2:** Proportion of mixtures 1:6 by volume for 1 m^3^

Mixtures	Cement (kg)	Sand (kg)	CC-BA (kg)	Water (L)
CPII-00	143.5	860.1	-	209.8
CPII-05	142.2	852.9	7.1	210.2
CPII-10	141	846.8	14.1	209.9
CPII-15	140.2	840.8	21	208.5
CPII-20	139	833.6	27.8	208.5
CPII-25	138.1	828.2	34.5	206.6

The experimental program comprised comprehensive characterization of the raw materials, as well as evaluation of the mortars in both fresh and hardened states. The constituent materials were characterized by X-ray fluorescence for chemical composition, XRD analysis, particle size distribution analysis, apparent density, and morphological assessment by scanning electron microscopy. The fresh mortars were evaluated through consistency index testing, density, incorporated air content determination, rheological analysis for squeeze-flow, and isothermal calorimetry test. In the hardened state, the mortars were subjected to flexural mechanical strength and tensile bond strength tests, in addition to water absorption by immersion and void index measurements. Complementary microstructural characterization of the hardened mortars was performed using scanning electron microscopy, X-ray diffraction, and micro-computed tomography, enabling correlation between microstructural features and the observed physical and mechanical performance.

The experimental program was designed to evaluate the applicability of the ash in simple construction systems, particularly those commonly adopted in the Brazilian context. Accordingly, the proposed mixtures were formulated to reproduce realistic field conditions in which the use of chemical admixtures is often avoided due to cost constraints.

Table 3 X-ray fluorescence (S2 Ranger, Bruker) was performed to determine chemical compositions, and the results are shown in Table 3. In this analysis, approximately 72% of the composition of CC-BA is predominantly calcium oxide (23.99%), followed by sodium oxide (18.6%), magnesium oxide (15.8%), and silica (13.21%). Meanwhile, the composition of the cement is primarily composed of calcium oxide (61.59%), silica (13.21%), and alumina (4.06%). According to the Brazilian standard (ABNT 2012), for a material to be classified as pozzolanic, the sum of SiO_2_ + Al_2_O_3_ + Fe_2_O_3_ must be greater than or equal to 50%. For CC-BA, this sum is 23.25%, indicating the absence of pozzolanic activity. However, ash can have a filler effect or heterogeneous nucleation, which can improve the rheological and mechanical properties of mortars (Farinha et al. 2018; Martirena and Monzó 2018).

**Table 3:** Chemical composition of precursors

	CaO	Na_2_O	MgO	SiO_2_		SO_3_	Fe_2_O_3_	K_2_O	Al_2_O_3_	Cl	Others	LOI
CC-BA (%)	23.99	18.6	15.8		13.21	6.64	5.77	5.20	4.27	3.23	3.13	13.17
Cement (%)	61.59	-	1.84		17.95	3.09	3.25	0.71	4.06	-	0.36	9.65

The oxides identified in this analysis are consistent with the peaks observed in the X-ray diffraction (XRD) pattern of the ash, where the most intense peaks correspond to the most abundant oxides. The Na_2_O and Cl content detected in this test may be associated with the saline environment of the coastal regions where the material is found. The chemical composition of CC-BA was similar to the biomass ash results (Farinha et al. 2018), particularly due to the considerable Cl content (6.54%). However, it differed from the predominant chemical composition found in coal ash from thermoelectric power plants (Khupsare et al. 2024), which contained 55.34% silica, 26.86% alumina, and 5.76% ferric oxide.

Although the ashes present relatively high levels of SO_3_ and alkalis (sodium and potassium) according to NBR 12653 (ABNT 2012), the percentage incorporated into the mortars is small in relation to the total mass and does not represent a significant amount capable of compromising performance. Furthermore, no signs of efflorescence or cracking associated with these compounds were observed during the experimental period.

**Fig. 6:**
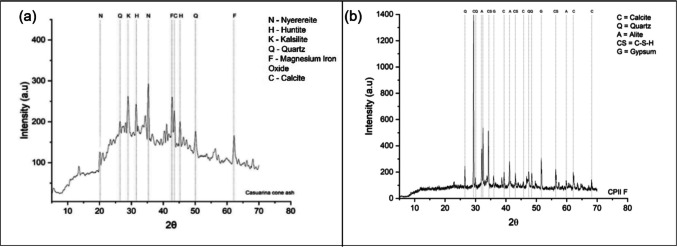
XRD of **a** CC-BA after grinding and **b** cement

**Fig. 7:**
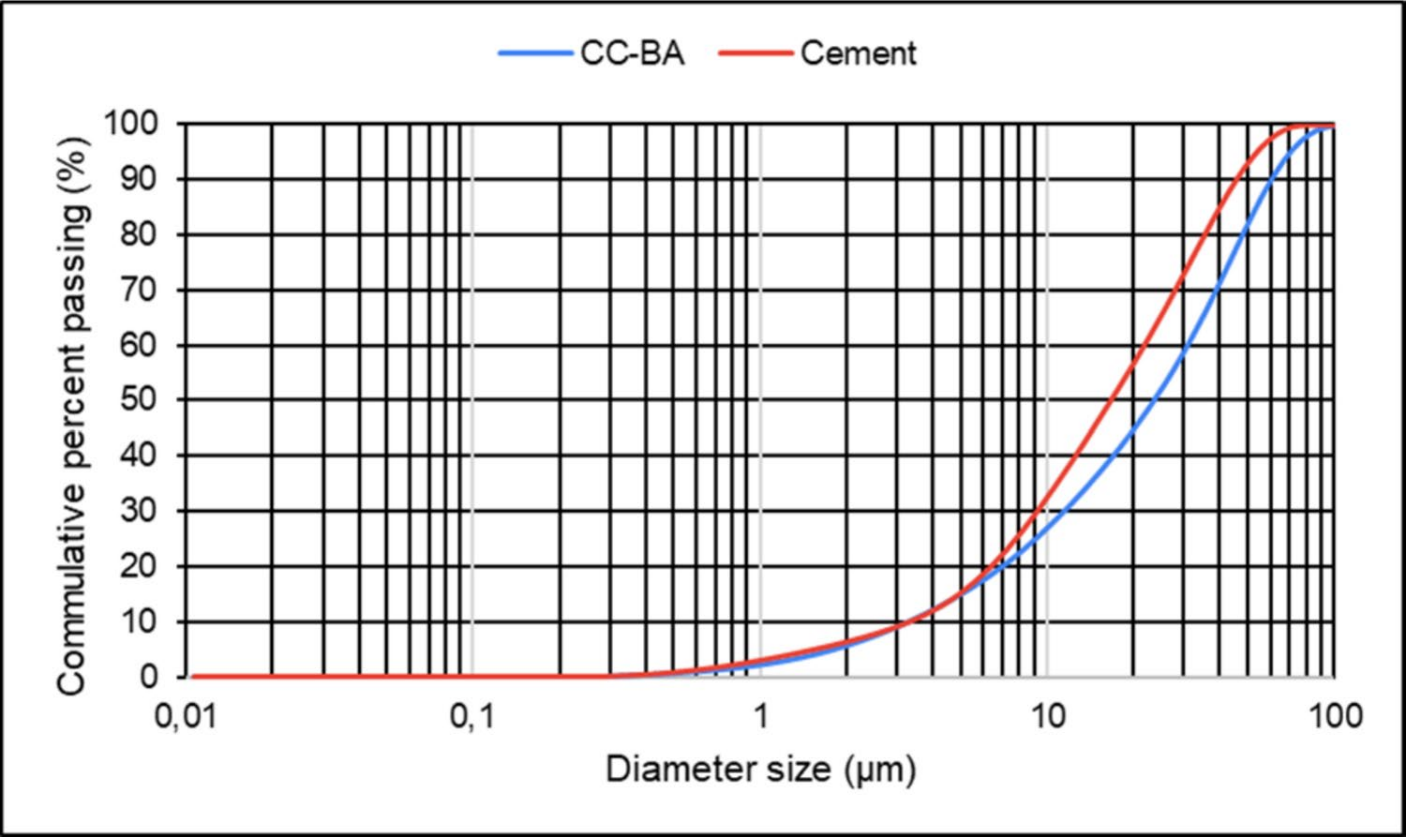
Laser granulometry of CPII-F and CC-BA

The compounds identified by XRD analysis (Miniflex, Rigaku) mostly have peaks corresponding to Na_2_Ca(CO_3_)_2_ (nyerereite), SiO_2_ (quartz), Mg_3_Ca(CO_3_)_4_ (huntite), and (MgO)(FeO) (magnesium iron oxide) (Fig. 6). An amorphous halo observed between 20 and 27° indicates a non-crystalline phase that contributes to the formation of reactive products, particularly promoting the development of calcium silicate hydrate (C-S-H) gel during cement hydration. This amorphous fraction may also facilitate heterogeneous nucleation of finer particles, as evidenced by granulometric analysis showing that 6% of the clay fraction is < 2 μm (Fig. 7). Similar to the results observed for CC-BA, the analysis of the cement (Fig. 6b) is also consistent with the XRF findings, predominantly exhibiting calcite, followed by quartz and alite (C_3_S).

The mineralogical composition of CC-BA is strongly influenced by cultivation conditions, including soil type and fertilizer application (Ayobami 2021). Elevated levels of Na and Cl in CC-BA are likely related to the soil characteristics where the trees were grown. Although the nyerereite peak may not directly represent the pure compound, it could be associated with soda ash, a material historically abundant and exploited in the region of cone collection. In contrast, low-quality biomass ashes predominantly display crystalline phases, which limit their pozzolanic reactivity (Martirena and Monzó 2018).

It determines the particle size distribution (Malvern Panalytical, Mastersizer 3000), where it is possible to observe that from *D*_10_ to approximately *D*_16_, the ash and cement exhibit overlapping particle size ranges (Fig. 7). Before this range, approximately 6% (clay fraction < 2 μm) consists of particles as fine as cement. Although this value does not correspond to the mean diameter, it is significant and indicates the influence of the incorporation of finer CC-BA particles on the subsequent results. 

At the particle size dimension, *D*_50_= 23.08 μm for CC-BA and *D*_50_= 17.39 μm for cement indicate the influence of the sieving process at 75 μm, which was performed to minimize discrepancies in particle fineness between the materials (Silva et al. 2022). At *D*_90_, the particle size distribution results show CC-BA *D*_90_ = 58.99 μm and cement *D*_90_ = 48.62 μm, representing the upper bound of the particle size distribution, which may influence the mixture.

The finer particles of the ash also contribute to the material’s ability to form reactive compounds, such as C-S-H, derived from ash hydration, which promotes a reduction in voids and greater compaction (Cheng et al. 2022). This can be observed in the increased flexural strength of the mortars.

Thus, despite the similarity in particle size between cement and ash, it is noted that the fineness of the cement is slightly higher than that of the ash. According to the other results, particularly in terms of mechanical strength and workability, the granulometry contributes to the filler effect and suggests behavior akin to reactive particles.

Based on the specific mass analyses conducted using the Le Chatelier method for cement and ash and the pycnometer method for sand, CC-BA identified as the least dense material (Table 4). This behavior is attributed to its porous particles, as evidenced by morphological micrographs below (SEM). Such low density contributes to the reduced weight of the mortar; however, it also increases water demand (Mangi et al. 2018).

**Table 4:** Results of apparent density

Materials	Density (g/cm^3^)	Standards
CC-BA	246	NBR 16605 (ABNT, 2017)
CPII-F	287	NBR 16605 (ABNT, 2017)
Sand	271	NBR 6457 (ABNT 2016)

The cement representing the densest component in the mortar mixture (Gencel et al. 2021) plays a key role in mechanical strength. When combined with ash additions at appropriate dosages, it enhances workability and reduces overall cement consumption (Nayak et al. 2022).

Sand exhibits an intermediate density and, as an inert constituent, plays a significant role in the overall density of the mortar matrix. Accordingly, its influence on fresh-state properties is mainly governed by physical parameters such as particle packing, void content, and apparent density (Carasek et al. 2016). As the sand type and content were kept constant across all mixtures in the present study, its contribution remained unchanged and therefore does not account for the observed differences, which are instead attributed to variations in the composition of the other constituents.

**Fig. 8:**
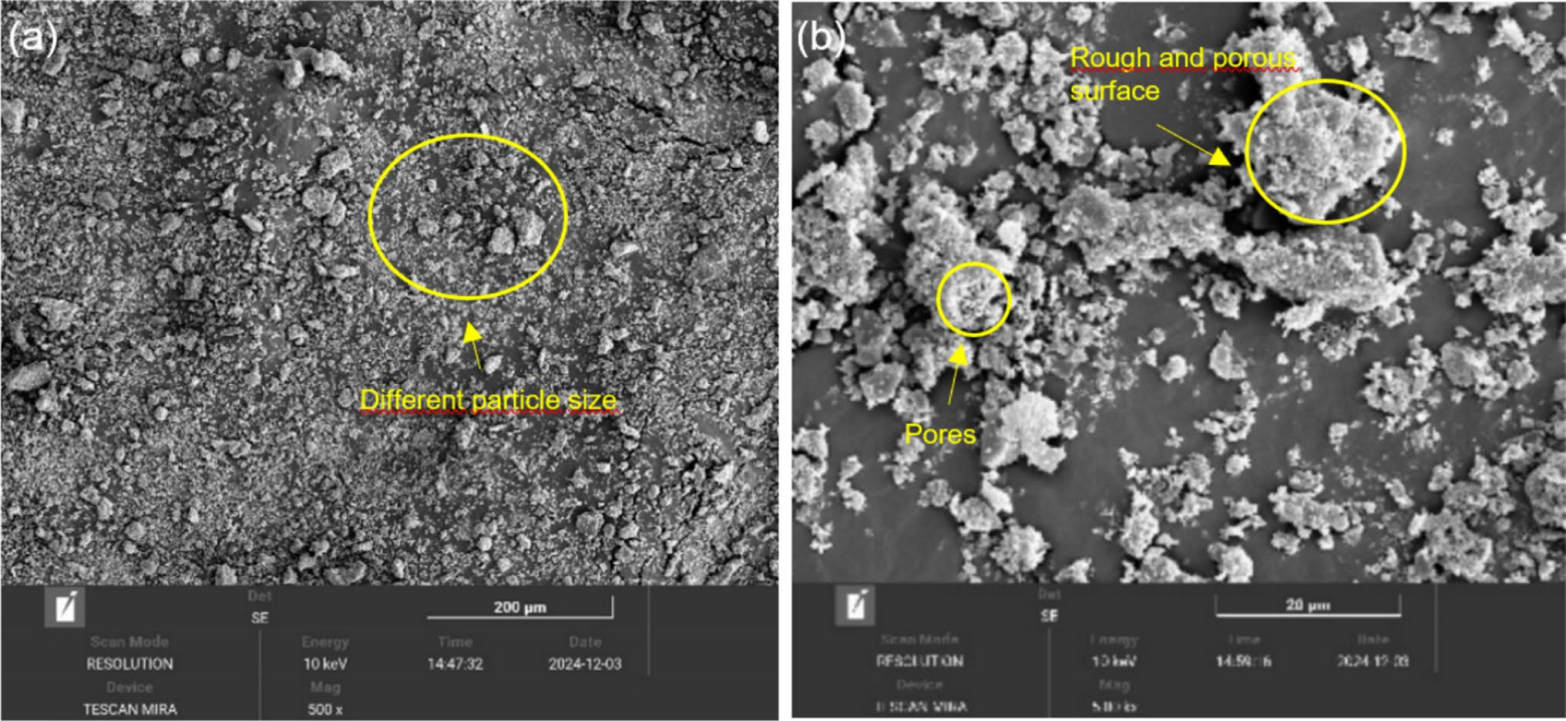
SEM CC-BA **a** 500x and **b** 5000x

Scanning electron microscopy (SEM) images of CC-BA reveal morphological features such as particle size irregularities (Rashad 2016), rough and porous surfaces (Gencel et al. 2021), and varied particle shapes (Srivastava and Singh 2020) (Fig. 8). Similar characteristics are observed in wood ash, which may negatively affect the workability of the mixture due to its high water absorption capacity, associated with an increased specific surface area (Zhuge et al. 2022).

The pozzolanic activity index was determined in accordance with NBR 15895 (ABNT 2010), and the obtained value was 80.25 mg Ca(OH)_2_/g. A material is classified as pozzolanic when the CaO consumption exceeds 330 mg CaO/g (equivalent to 436 mg Ca(OH)_2_/g of pozzolan). Therefore, CC-BA is characterized as non-pozzolanic. This conclusion is supported by the XRF analysis, which indicates a comparatively higher CaO content in the cement than in CC-BA, which may contribute to the absence of pozzolanic reactivity. Furthermore, according to the Luxán method, the pozzolanic activity result was also negative.

## Results and discussion

Table 5 shows the consistency index according to the Brazilian standard NBR 13276 (ABNT 2016) and the proportions; the spreads must meet the requirement of 260 ± 5 mm as the maximum and minimum spread limits for the mortar.

**Table 5:** Spreading, water/cement ratio, and water/dry material ratio

Consistency index (mm)	Water/dry materials ratio	Water/cement
260	0.209	1.46
258	0.210	1.48
261	0.209	1.49
258	0.208	1.49
259	0.208	1.5
262	0.206	1.5

The water-to-dry-material ratio decreases with increasing ash content because the incorporation of ash increases the total volume of dry materials in the mixture, while the cement and sand contents remain constant. Consequently, for a fixed amount of mixing water, the relative proportion of water with respect to the total dry constituents is reduced. The fineness of the ash particles, derived from the milling process, requires a higher amount of water, which may improve workability (Oruji et al. 2017), as evidenced in the granulometric analysis. In coal ashes, the higher specific surface area increases the water demand of the mixture due to the greater amount of water required to wet the solid particle surfaces. However, several factors influence the increase or decrease in workability, including milling time, the source of the waste, and the material’s fineness (Al Biajawi et al. 2022).

In addition, the variations in the water-to-cement (w/c) and water-to-dry-material (w/dm) ratios among the mixtures were kept very small and did not significantly affect the consistency index.

**Fig. 9:**
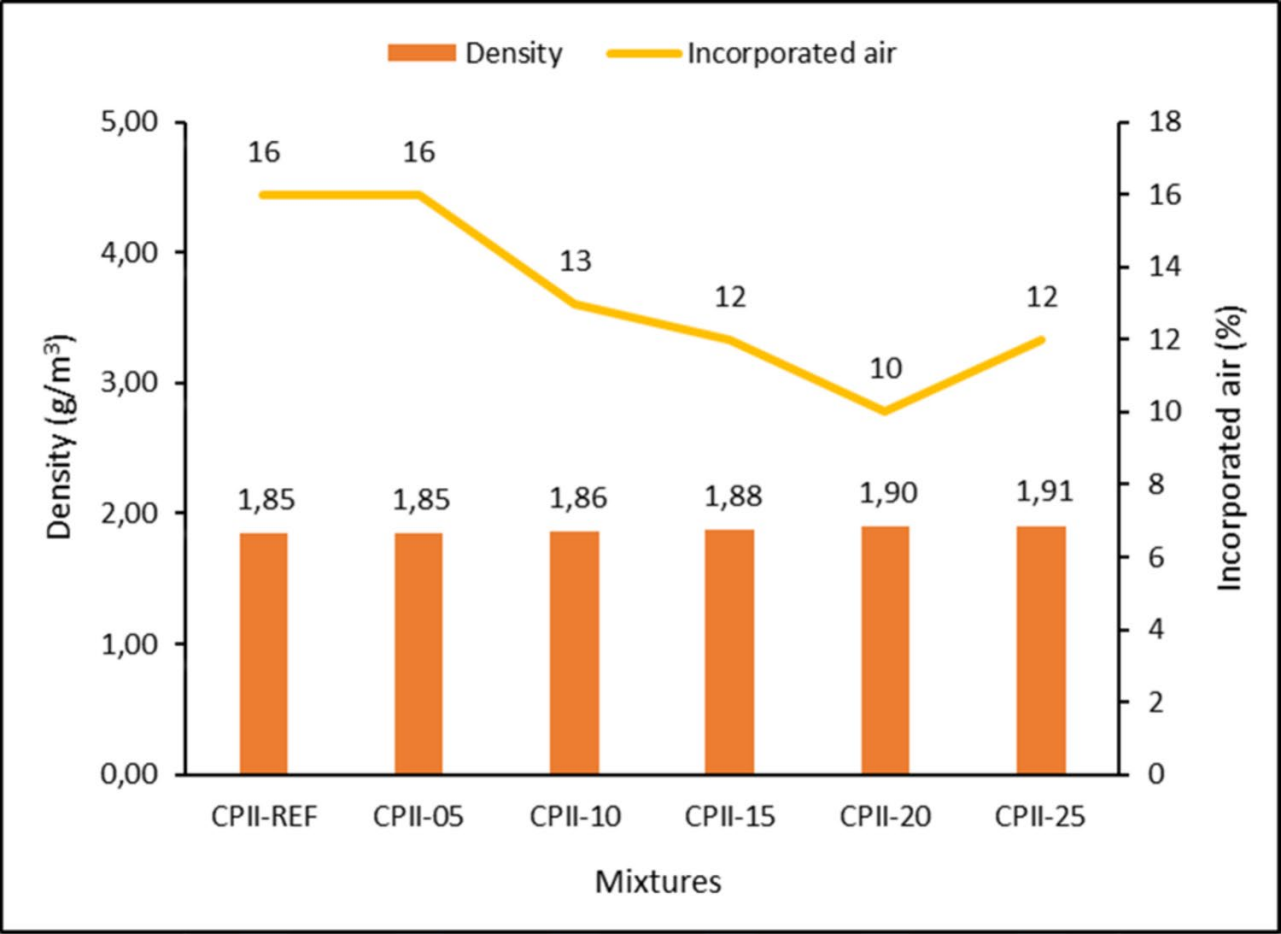
Density in fresh state and incorporated air

A trend of increasing density is observed, from 1.85 in the reference mortar to 1.91 in CPII-25, as the amount of CC-BA addition increases (Fig. 9). This can be attributed to the fact that no material is replaced, but rather, an additional element is incorporated into the mortar. However, the density values obtained are consistent with the ranges historically adopted in the Brazilian standard NBR 13281 (ABNT 2005a, b), which reported typical mortar densities on the order of 1.2–2.2 g/cm^3^. In the current version NBR 13281-1 (ABNT 2023), mortar density is expressed through classification ranges. For fresh mortars, the standard defines density classes from DF0 (< 1.4 g/cm^3^) to DF4 (≥ 2.0 g/cm^3^), the density values obtained are also satisfactory, while the measurement procedure used to obtain these values follows the method described in NBR 13278 (ABNT 2005a, b).

The reduction in the incorporated air content, from 16 to 10%, remains satisfactory, with a suggested maximum limit of up to 20% (Antoniazzi et al. 2020). The increase in density and the decrease in air content as CC-BA is added can be associated with rigid compaction, closing the existing porosity (Babajide Olabimtan and Mosaberpanah 2023), as evidenced by µCT (Fig. 17). Furthermore, as indicated by the particle size distribution analysis, the reduction in air content reflects the influence of the presence of finer ash particles, which promote the formation of hydration products and nucleation effects.

In ashes used as a substitute for cement, we observed an increase in the density of the ash, similar to CC-BA (Mangi et al. 2018), due to the finer particles requiring more water. However, in the same study, the formation of a porous structure and increase in the incorporated air content as the amount of ash increased diverged from CC-BA. In the present study, however, CC-BA was added as an additional material rather than replacing cement, and thus, the observed density behavior reflects a different mix design strategy directly equivalent.

**Fig. 10:**
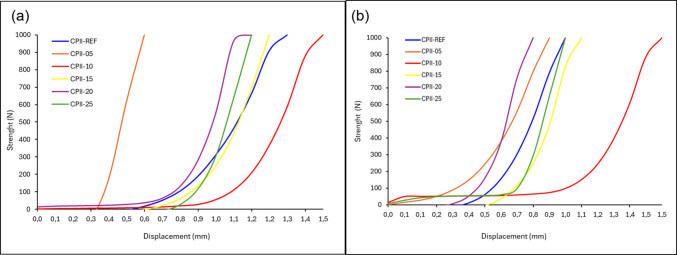
Squeeze-flow curves **a** 15 min and **b** 60 min

Figure 10 shows the squeeze-flow curves of the mixtures after 15 and 60 min of rest. After 15 min (Fig. 10a), a greater displacement is observed in the CPII-10 mortar, followed by the CPII-00 and CPII-15 mixes, indicating lower viscosity and higher workability, facilitating its spreading during application for coatings. The lower displacement for CPII-05, followed by CPII-20 and CPII-25, suggests characteristics of lower fluidity, which may occur when there is a high amount of fine particles or insufficient water in the mortar, as seen with the higher additions. Regarding CPII-05, this behavior can be associated with the portion designated for squeeze-flow, given the satisfactory results in the other tests. After 60 min (Fig. 10b), the mortar behavior remains similar with no significant changes in spreading (Nascimento et al. 2025), favorable for application due to workability. In practical terms, this means that the mortar can wait for 60 min before use without drying out.

Therefore, the liquid portion absorbed by the aggregates consists of water and binder, which may favor the hydration of the binders in the mortar and increase adhesion (Farinha et al. 2018). Other results consistent with the squeeze-flow include the tensile bond strength, where the best results were obtained in the two mixes mentioned above, and flexural strength, which also showed satisfactory results. The decrease in air content correlates with the greater force required for displacement, indicating a lower amount of voids in the mortar.

For coating mortars, a moderate flowability is required, so that spreading is not hindered by high viscosity, nor is there material loss due to excess water (Ribeiro et al. 2021).

**Fig. 11:**
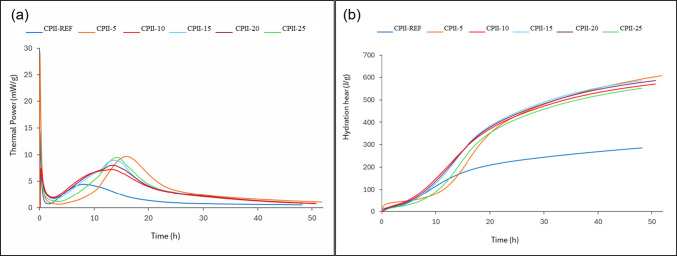
Results of calorimetric **a** thermal power and **b** hydration heat

The results of the isothermal calorimetry test (Calmetrix I-CAL 2000), presented by the released heat (Fig. 11b) and total accumulated heat (Fig. 11a), were conducted according to the guidelines of the American standard C1679 (ASTM 2017). The mixture of cement, water, and, eventually, ash is homogenized externally to the equipment, allowing the initial hydration heat to be released and detected immediately, justifying the higher peak in the first few minutes, during the dissolution period.

Which varied between 1 and 2 h for CPII-00, this same stage occurred with variations between 2 and 4 h for CPII-15, CPII-20, and CPII-25, and between 6 and 8 h in the heat flow for CPII-05 and CPII-10 percentages, delaying the setting time (Skevi et al. 2022), favoring higher workability, which implies the moldability of this mortar when applied to the wall.

The heterogeneous nucleation of the finer particles occurs, forming amorphous C-S-H. This reaction proceeds as expected, in agreement with the particle size distribution results, where 6% of particles smaller than 2 μm were identified in both CC-BA and CPII-F. Therefore, the acceleration of released heat was higher in all cases with the addition of CC-BA. Notably, in the hydration heat, CPII-05 and CPII-15 showed the highest peaks, indicating that more stable phases, different from portlandite (Ca(OH)_2_), are formed in the mixture.

The deceleration of the hydration phase formation occurs between 13 and 16 h for the mixtures with additions, while the same process begins at 8 h for CPII-00, indicating slower hydration reactions with CC-BA compared to CPII-F.

Ultimately, it is characterized by the transformation of ettringite (AFt) into monosulfate (AFm), and these reactions occur slowly (Ribeiro et al. 2021). Additionally, there is an increase in strength during the final hydration phase of the mortar, which can be confirmed by the mechanical test results.

The greater heat release observed in mixtures containing CC-BA is associated with a physical effect related to heterogeneous nucleation. The fine particles of CC-BA provide additional surfaces for C-S-H nucleation, thereby promoting accelerated cement hydration kinetics and increasing the initial heat evolution. Accordingly, the observed behavior is interpreted as a physical effect driven by nucleation, rather than as evidence of supplementary cementitious chemical reactivity. In addition, as the CC-BA content increases, the water demand of the mortar rises proportionally, due to the high initial hydration rate (Tayeh et al. 2019).

**Fig. 12:**
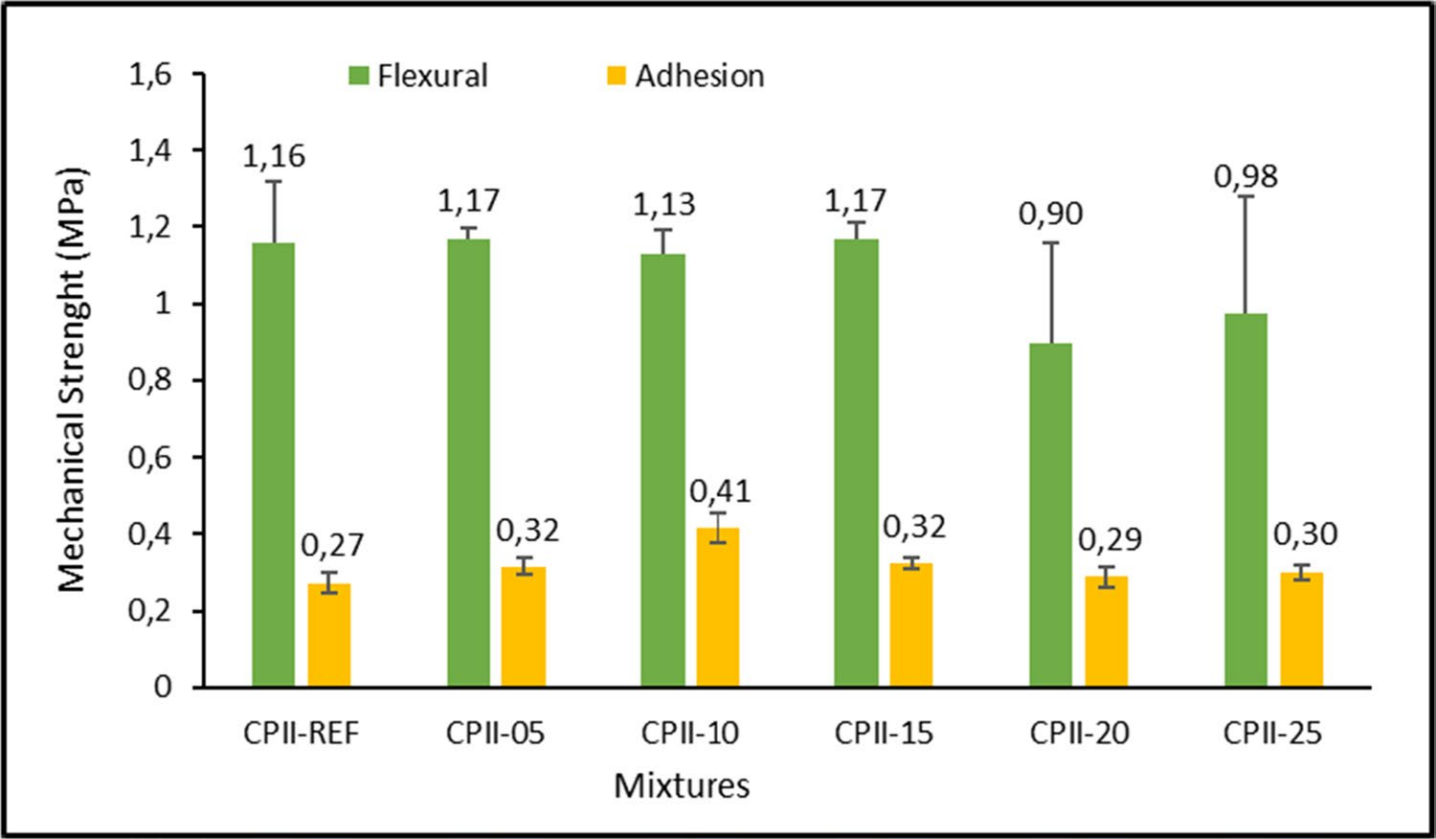
Mechanical strength mortars

Figure 12 shows the best performances in terms of flexural strength and adhesion for the average mixtures of CPII-05, CPII-10, and CPII-15, which are among the best results achieved in accumulated heat release during hydration and rheology. It can be understood that the more stable phases formed in the mixture directly contribute to its mechanical strength up to CPII-15.

The Brazilian standard NBR 13282-1 (ABNT 2023b) sets minimum and maximum values for flexural tensile strength ranging from 1.0 to 4.5 MPa and for potential tensile bond strength between < 0.20 and ≥ 0.30. Thus, with the exception of the CPII-20 and CPII-25 mixtures in the flexural test, all values meet the standard. The adhesion values found in CPII-05, CPII-10, and CPII-15 even met the criteria for mortars applied in external environments that receive coatings or are exposed to more severe environmental conditions, according to the standard.

The mixtures CPII-20 and CPII-25 showed a decrease in all mechanical strength tests related to the water content in the mixture, as observed in the consistency index, which, after curing, tends to form voids. The addition of non-pozzolanic or low-reactive material, as observed in XRF and XRD, also contributes to the decrease in mechanical strength due to the lack of formation of hydration-related compounds such as C-S-H, CH (portlandite), and ettringite. Larger additions are also associated with the consequent reduction of hydration reactions in CPII-F, resulting from the lower amount of this material in the mortars, which impacts the lower production of pozzolanic reactions, as well as the decline in flexural strength (Chindasiriphan et al. 2023).

The decrease in mechanical strength as the addition content increases corroborates with the porosity observed in the SEM results of the mortars (Carević et al. 2019). Substitutions above 15% typically compromise the durability and strength of mortars with ash additions (Jones et al. 2023).

The adhesive strength of CPII-10 demonstrated better performance compared to the other compositions, consistent with the calorimetry analysis, where the more intense heat peak was identified earlier, highlighting heterogeneous nucleation, where the formation of C-S-H around the finer particles is promoted. Furthermore, the fine particles occupy the voids left between the cement and sand grains, as seen in the decrease in void index for this mixture, generating the filler effect. It should be noted that CC-BA was not proposed as a cementitious addition with pozzolanic function, but as a predominantly inert material incorporated at low dosage, acting mainly through physical mechanisms such as filler effect and heterogeneous nucleation.

The results obtained in the squeeze-flow test also align with the adhesion, as, generally, the primary relationship of the rheological behavior of the mortars lies in their adhesive tensile strength when applied to the substrate and the spreadability of the mortar (Stolz et al. 2016).

**Fig. 13:**
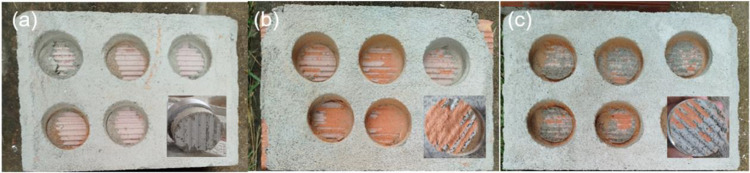
Thickening time for samples **a** CPII-00, **b** CPII-10, and **c** CPII-15

The adhesion tests exhibited visual characteristics of good adhesion and cohesion to the CPII-10 and CPII-15 substrates, surpassing even the reference mortar (Fig. 13). It was possible to observe the mortar’s fixation to the substrate after pull-off, with no cracks and remaining intact. The combination of these cohesion and adhesion characteristics in balance, in other words, the fixation to the substrate combined with the mortar’s ability to remain intact, indicates a satisfactory performance of the mortar. The squeeze-flow results also align with this outcome, where the primary relationship of the rheological behavior lies in its tensile adhesion strength when applied to the substrate and the spreadability of the mortar (Chindasiriphan et al. 2023).

By comparing the statistical analyses from ANOVA with the standard deviations, it can be observed that, for flexural strength, despite the considerable standard deviation, the difference between the means is not significant, leading to some inconsistency in the information (Table 6). However, when adhesion is considered, there is an indication of a difference upon the addition of CC-BA, highlighting the significance of CC-BA incorporation, which is particularly pronounced at a 10% addition, resulting in a notable increase in adhesion.

**Table 6:** DIC- ANOVA (*p* ≤ 0.05) for flexural strength and adhesion at 28 days

Test	Source	DL	MS	QM	*F*_cal_	*F*_tab_	Significance
Flexural (MPa)	Treatment	5	0.192	0.038	1.06	3.11	N.S
	Residual	12	0.187	0.036	-	-	
Adhesion (MPa)	Treatment	5	0.024	0.0048	4.80	3.11	*
	Residual	12	0.012	0.0010	-	-	

*Significant

**Fig. 14:**
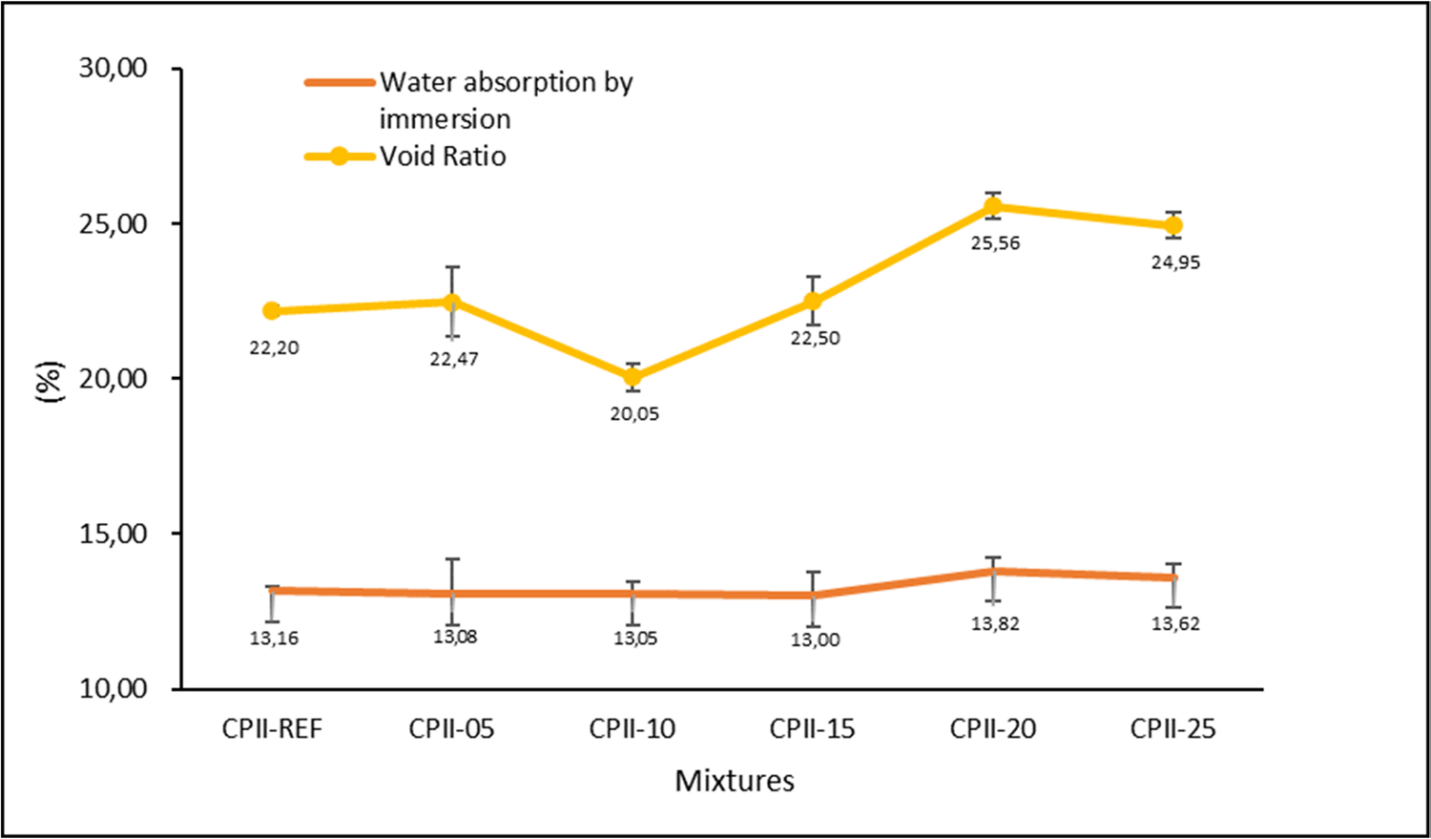
Results of water absorption by immersion and void ratio

The water absorption by immersion and void index are directly interconnected, where up to CPII-15, there was a reduction or similarity in the results compared to CPII-00, indicating compaction and pore closure (Fig. 14). It is noteworthy that, for water absorption, the standard deviations are coincident, meaning that statistically, the results do not show significant differences but rather demonstrate a trend of reduction in CPII-10 and CPII-15. Meanwhile, for CPII-20 and CPII-25, the increase observed indicates an excess of CC-BA addition, which leads to an increase in porosity rather than its closure (Medina et al. 2019).

Employing a completely randomized design (CRD) at a 5% significance level (*p* ≤ 0.05), the results indicate that the mean values for water absorption by immersion do not differ significantly (Table 7). In contrast, the analysis reveals that the void index exhibits statistically significant differences between means, suggesting that the incorporation of CC-BA may have influenced these outcomes. This observation can also be associated with the entrained air content, where, except for the 5% CC-BA addition, all other levels displayed reduced values, likely reflecting the void-filling effect induced by CC-BA incorporation.

**Table 7:** CRD- ANOVA (*p* ≤ 0.05) for water absorption by immersion and void ratio at 28 days

Test	Source	DL	MS	QM	*F*_cal_	*F*_tab_	Significance
Water absorption (%)	Treatment	5	0.678	0.136	1.032	3.11	N.S
	Residual	12	1.577	0.131		-	
Void ratio (%)	Treatment	5	0.392	0.078	4.102	3.11	*
	Residual	12	0.229	0.019		-	

*Significant

**Fig. 15:**
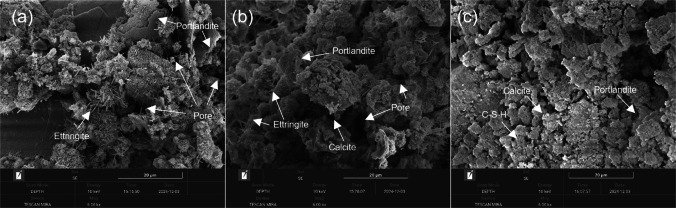
SEM of mortars after 28 days **a** CPII-00, **b** CPII-10, and **c** CPII-15

Figure 15 shows as the addition content increased, compared to CPII-00, a decrease in the appearance of ettringite and a denser structure were observed (Huynh and Ngo 2022), although still porous, which is related to the morphology of the ashes and unburned coal. This observation aligns with the μ-CT results, where a considerable number of larger pores are visible, but a smaller percentage of the total pores compared to CPII-00. The presence of ettringite is clearly identified in CPII-00, whereas in the mixtures with additions, its presence is less evident, especially in CPII-15. Due to the chemical composition of CC-BA, which predominantly contains CaO, similar to cement, the products formed such as C-S-H, portlandite, and calcite can also be associated with the contribution of CC-BA, with its presence being more noticeable in mortars with some percentage of addition (Oruji et al. 2017). In contrast, since this analysis was conducted on a small fragment of the test specimen, some products may be more or less evident.

**Fig. 16:**
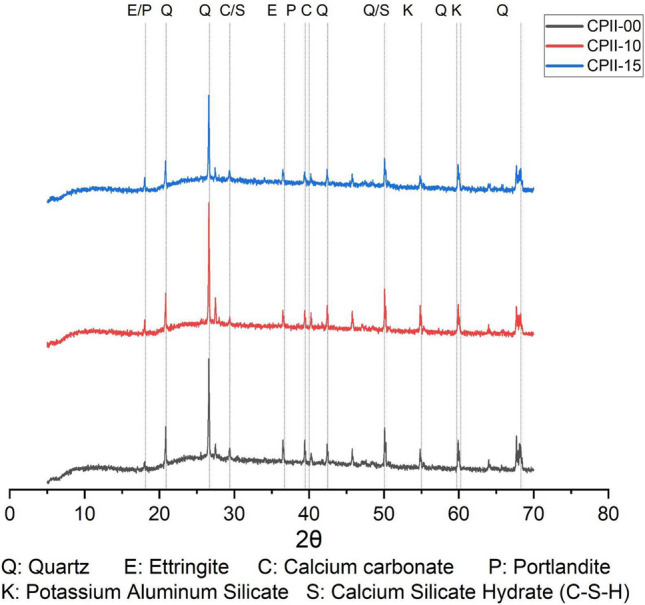
DRX of mortars after 28 days CPII-00 CPII-10 and CPII-15

There is a predominance of quartz peaks, responsible for the density and stability of the mortar (Fig. 16). The ettringite peaks indicate the hydration of tricalcium aluminate (C_3_A), with expansive characteristics that contribute to the closure of porosity. The mortars with CC-BA addition did not visually show any cracking up to 28 days of curing, contrary incineration bottom ash (Huynh and Ngo 2022). Calcium carbonate and portlandite are typically associated with the carbonation process (Nayaka et al. 2018). Potassium and aluminum silicate are typically present in bottom ashes; its presence may indicate physical reactions and the formation of C-S-H, which contributes to durability and increased mechanical strength. The reduction in the intensity of the portlandite peak from CPII-00 to CPII-15 may occur with the formation of amorphous C-S-H derived from the heterogeneous nucleation of fine particles, enhancing mechanical strength and reducing the void index up to CPII-10, as well as the more intense heat peak in the early hydration reactions.

Due to the high quartz content originating from the aggregate fraction, the XRD patterns limit the resolution of minor crystalline phases. Therefore, the diffractograms are interpreted primarily as qualitative, supportive evidence and not as a standalone quantitative phase analysis.

Analyzing the data contained in the percentages of closed and open porosity, the respective values are 2.506% and 8.792% for the reference, 2.354% and 3.709% for CPII-10, and 3.102% and 5.582% for CPII-15 (Table 8). Thus, the closed and open porosity of CPII-10 was the lowest compared to the others. Furthermore, the total porosity of the additions was lower compared to CPII-00, with the lowest values concentrated in CPII-10, even surpassing CPII-00. Despite having larger pores, the mortars with additions have a lower pore volume, which contributes to the mechanical strength gain due to the filler effect, as evidenced by the density and air content results. In contrast to the addition of CC-BA, presents results showing an increase in total porosity, although also indicating some filling (Vaičienė et al. 2023).

**Table 8:** Results obtained from μ-CT analysis

Description	CPII-00	CPII-10	CPII-15
Number of closed pores (units)	18.288	9.250	25.837
Volume of closed pores (mm^3^)	2.084	2.067	2.671
Surface area of closed pores (mm^2^)	155.270	98.552	131.260
Closed porosity (%)	2.506	2.354	3.102
Open porosity (%)	8.792	3.709	5.582
Total volume of pore space (mm^3^)	10.100	5.449	7.760
Total porosity (%)	11.077	5.976	8.511

**Fig. 17:**
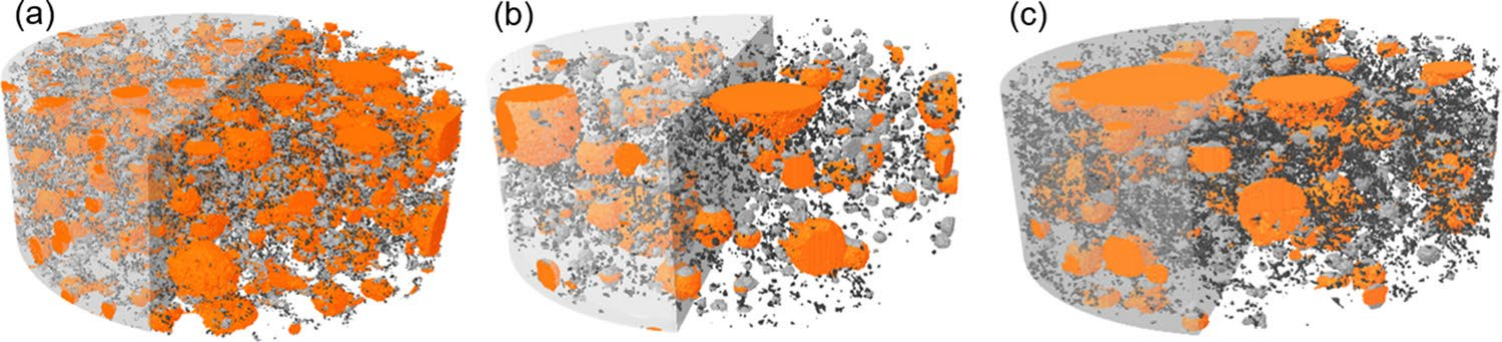
μ-CT 3D image of mortars after 28 days **a** CPII-00, **b** CPII-10, and **c** CPII-15

The 3D images illustrate the distribution of small (*p* < 0.05 mm), medium (0.05 < *p* < 0.13 mm), and large pores (*p* > 0.13 mm) in the samples (Fig. 17). Quantitative results from μ-CT analysis (Table 8) show that CPII-15 presents the highest closed porosity (3.102%), compared with 2.506% for CPII-00 and 2.354% for CPII-10. In contrast, CPII-00 exhibits the highest open porosity (8.792%), followed by CPII-15 (5.582%) and CPII-10 (3.709%). These differences are consistent with the trends observed in density, air content, and water absorption by immersion.

When comparing the substitution of 5% wood ash with the reference mortars in the study by Nascimento et al. (2025), we obtained contrary results for open porosity percentage and the number of closed pores, compared to the CC-BA mixtures of CPII-00 with CPII-10. Specifically, mixtures with wood ash showed a greater number and size of pores, while CC-BA systems showed a more refined pore structure. Despite these differences, both materials promoted a filling effect within the cementitious matrix, which contributes to microstructural densification. This suggests that, although the mechanisms influencing pore structure are not identical, the addition of fine particulate residues can increase particle packing and modify porosity through a similar physical action.

## Conclusions

This research has developed a novel perspective and source for obtaining bottom ash, demonstrating through conducted experiments the feasibility of its application as an possible filler addition in mortars for walls and ceilings.

The bibliometric survey highlights a clear scarcity of studies on the application of *Casuarina equisetifolia* in construction materials, particularly in coating mortars, revealing a significant gap in the literature. By providing systematic experimental evidence on the physical, mechanical, and microstructural performance of mortars incorporating CC-BA, the present study addresses this gap, expands the existing knowledge base, and advances sustainable waste valorization strategies in construction materials.

The characterization of the ash, along with the other materials, aimed to assess the similarity between CC-BA and cement, with a particular emphasis on particle size distribution analysis. Although the material did not exhibit pozzolanic activity and the combined content of SiO_2_, Al_2_O_3_, and Fe_2_O_3_ was low for this effect (23.25%), it clearly demonstrated improvements compared to CPII-00, either due to its filler effect or hydration reactions.

The fresh-state analyses demonstrated satisfactory results up to CPII-15, with CPII-10 standing out. In the consistency index test, all proposed mixtures complied with the standard; however, a higher water demand was required as the residue content increased. This outcome influenced the rheology of the mortars, highlighting improved workability and viscosity for CPII-10, followed by CPII-15, with no significant differences in load versus displacement between 15 and 60 min of testing. In calorimetry analysis, the best results in terms of accumulated heat and heat flow were observed for CPII-05 and CPII-15, reinforcing the hypothesis of optimal addition levels up to 15%. The bulk density and air content tests also yielded expected results, despite the increase in density due of the added material. Meanwhile, the incorporated air content in all modified mixtures was lower than in CPII-00, indicating a filler effect.

In the hardened state, after 28 days, the mechanical strength of the mortars stood out, with results not only meeting but exceeding standard requirements, particularly for CPII-10. The water absorption by immersion and void index results also showed an increase compared to CPII-00 for mixtures with CPII-20 and CPII-25 additions, while percentages up to CPII-15 were lower or close to the reference.

The microstructural analyses of the mortars, particularly through X-ray microcomputed tomography, confirmed the findings observed in both the fresh and hardened states, indicating a filler effect that enhanced properties up to CPII-15.

In conclusion, CPII-10 was the percentage that best met the proposed criteria; however, CPII-15 may also be acceptable for application in walls and ceilings based on the results of mechanical strength, water absorption by immersion, calorimetry, and incorporated air content; however, it requires validation against additional requirements. CPII-20 and CPII-25 demonstrated the negative effects of excessive supplementary material, impairing the necessary reactions in mortars. Therefore, these percentages are not suitable for rendering mortars for walls and ceilings.

## Data Availability

The datasets generated and analyzed during the current study are original and exclusively produced by the authors. Due to the involvement of proprietary material, part of the data cannot be made publicly available at this time. A portion of the content has been legally deposited as a National Patent Application, Utility Model, Certificate of Addition, and entry into the national phase of the PCT under the Process Number BR 10 2024 024545 8. Any additional data supporting the findings of this study may be provided by the corresponding author upon reasonable request, in compliance with intellectual property and confidentiality restrictions.

## Declarations

### Clinical trial number

This is not applicable.
